# Nucleation and dissolution mechanism underlying amyotrophic lateral sclerosis/frontotemporal lobar dementia-linked fused in sarcoma condensates

**DOI:** 10.1016/j.isci.2023.106537

**Published:** 2023-03-31

**Authors:** Nathalie A. Djaja, Matthew T. Chang, Freya R. Beinart, Vivian M. Morris, Laura R. Ganser, Sua Myong

**Affiliations:** 1 Program in Cellular Molecular Developmental Biology and Biophysics, Johns Hopkins University, 3400 N. Charles Street, Baltimore, MD 21218, USA; 2 Department of Biophysics, Johns Hopkins University, 3400 N. Charles Street, Baltimore, MD 21218, USA; 3 Department of Biology, Kenyon College, 106 College Park Dr, Gambler, OH 43022, USA; 4 Lymphoid Malignancy Branch, National Cancer Institute, National Institutes of Health, Building 10, Bethesda, MD 20814, USA; 5 T.C. Jenkins Department of Biophysics, Johns Hopkins University, 3400 N. Charles Street, Baltimore, MD 21218, USA

**Keywords:** Properties of biomolecules, Molecular interaction, Biophysics

## Abstract

Fused in sarcoma (FUS) is a nuclear RNA-binding protein. Mutations in FUS lead to the mislocalization of FUS from the nucleus to the cytosol and formation of pathogenic aggregates in neurodegenerative diseases including amyotrophic lateral sclerosis (ALS) and frontotemporal lobar dementia (FTLD), yet with unknown molecular mechanisms. Using mutant and stress conditions, we visualized FUS localization and aggregate formation in cells. We used single-molecule pull-down (SiMPull) to quantify the native oligomerization states of wildtype (WT) and mutant FUS in cells. We demonstrate that the NLS mutants exhibited the highest oligomerization (>3) followed by other FUS mutants (>2) and WT FUS which is primarily monomeric. Strikingly, the mutant FUS oligomers are extremely stable and resistant to treatment by high salt, hexanediol, RNase, and Karyopherin-β2 and only soluble in GdnHCl and SDS. We propose that the increased oligomerization units of mutant FUS and their high stability may contribute to ALS/FTLD pathogenesis.

## Highlights

• FUS wildtype in neuroblastoma cells is primarily monomers in the nucleus
• ALS/FTLD-linked FUS mutants show increased oligomers up to 5–6 units per cluster
• Despite increased FUS puncta under sorbitol stress, oligomers remain unchanged
• FUS mutant oligomers are extremely stable; they persist harsh chemical treatments

## Graphical abstract

**Figure:**
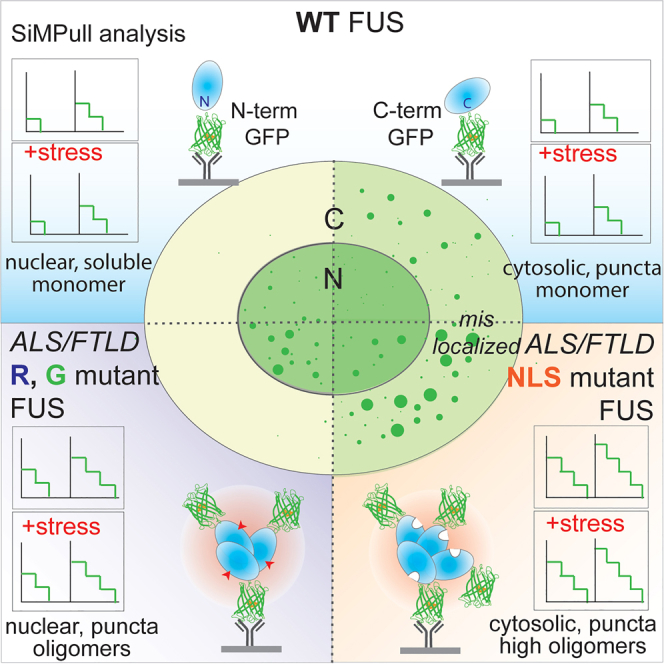

## Introduction

Liquid-liquid phase separation (LLPS) underlies the formation of biomolecular condensates in cells which helps to spatially organize and sequester specific cellular components for efficient processing.^1^^,^^2^^,^^3^^,^^4^^,^^5^^,^^6^ LLPS is governed by multivalent interactions of proteins and nucleic acids which form intra- and intermolecular interactions.^7^^,^^8^^,^^9^^,^^10^^,^^11^^,^^12^^,^^13^ Multivalent interactions promote high concentrations of macromolecules and drive rapid transitions from single molecules of proteins and nucleic acids to large oligomeric ribonucleoprotein (RNP) complexes.^14^^,^^15^ The mechanisms underlying LLPS have been widely studied as aberrant condensate formation is implicated in neurodegenerative diseases. Fused in sarcoma (FUS) is a nuclear RNA-binding protein (RBP) involved in many cellular processes such as transcription, splicing, and mRNA export.^13^^,^^16^^,^^17^^,^^18^ FUS can undergo LLPS by forming transient multivalent interactions with RNA and other RBPs.^19^^,^^20^^,^^21^^,^^22^ FUS mutations have been linked to amyotrophic lateral sclerosis (ALS) and frontotemporal lobar dementia (FTLD) in which pathological FUS inclusions cause neuronal degeneration.^23^^,^^24^^,^^25^^,^^26^ Aberrant LLPS in ALS/FTLD-linked FUS mutants results in the formation of cytotoxic irreversible aggregates.^27^^,^^28^^,^^29^

Current tools for investigating LLPS measure the formation and material properties of phase-separated condensates and include fluorescence recovery after photo bleaching (FRAP), Forster resonance energy transfer (FRET), fluorescence correlation spectroscopy, optical tweezers, microrheology and turbidity assays.^30^^,^^31^ While such tools are useful for probing the global properties of condensates, they are blind to molecular-level details, such as the status of oligomeric RNP complexes which may be the building blocks for the larger assemblies of condensates in cells. To address this question, we employed the single-molecule pulldown (SiMPull) assay to measure the oligomerization status of FUS-RNA complexes in neuroblastoma cells (SH-SY5Y). SiMPull enables capturing and quantifying soluble protein complexes directly from cell lysates. Previously, SiMPull has been used to determine the oligomerization of protein complexes including the monomeric vs. dimeric states of mTOR kinase.^32^^,^^33^

We chose to investigate wildtype (WT) FUS and several ALS/FTLD-linked FUS mutants to test the cellular oligomerization status that may drive the formation of FUS granules and aggregation. While ALS/FTLD-linked FUS mutants have been characterized *in vitro* at a droplet level^34^ and in cellulo and *in vivo* at a condensate level,^35^ FUS mutants have not been studied at a single-molecule level in cells. We asked if cellular condensates consist of smaller units of soluble oligomers of proteins. In conjunction, we sought to assess if the oligomerization pattern is correlated with the propensity of condensate formation with and without stress. Using the SiMPull platform, we also interrogated the pulled-down complexes by applying a diverse set of dissolving reagents to examine what molecular interactions are required for sustaining the oligomers.

We demonstrate that while FUS wildtype exists primarily as monomers, ALS/FTLD-linked FUS mutants display higher oligomers. Although osmotic stress induced by sorbitol drives mislocalization and puncta formation of FUS in cells, the basal oligomers remain unchanged for both wildtype and FUS mutants, suggesting that the oligomers are inherent basal units of FUS in cells. Strikingly, the oligomers remain intact even when treated with harsh chemical conditions such as high salt, hexanediol, and treatment by RNase A and Karyopherin-β2, indicating highly stable FUS complexes. Together, our findings suggest that the inherently stable and higher order complexes of FUS mutants may contribute to pathogenic aggregation.

## Results

### Validation of wildtype and mutant fused in sarcoma-GFP constructs

**Figure 1:**
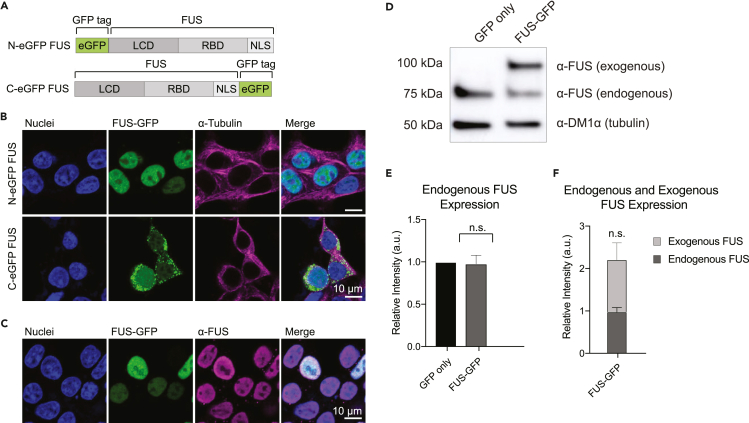
FUS-GFP expression in SH-SY5Y cells (A) GFP tag inserted in the N- or C-terminal region of FUS. (B) Immunofluorescent images of SH-SY5Y cells expressing N- or C-terminal GFP-tagged FUS. (C) Immunofluorescent images of cells expressing FUS-GFP and stained with FUS antibody. (D) Western blot of endogenous and exogenous FUS expression in cells transfected with GFP only (control) and FUS-GFP plasmids. (E) Relative quantification of endogenous FUS expression in GFP only and FUS-GFP transfected cells (N = 3, ∼30 cells/plasmid). (F) Relative quantification of endogenous and exogenous FUS expression in FUS-GFP transfected cells (N = 3, ∼30 cells/plasmid). Data information: In (E-F), data are presented as mean ± SD. ∗p ≤ 0.05 (One-way ANOVA).

We prepared green fluorescent protein (GFP)-tagged FUS constructs to assess the subcellular localization and FUS granule formation for WT and mutant FUS under normal and stressed cellular conditions. GFP tags were inserted at either the N or the C terminus of FUS (Figure 1A). Insertion of a GFP tag at the N terminus of FUS resulted in proper nuclear localization, consistent with endogenous FUS localization in the nucleus probed by immunofluorescence (Figure 1B top row, c). Conversely, the insertion of a GFP tag at the C terminus of FUS resulted in the mislocalization of FUS to the cytosol and appearance of FUS puncta in the cytosol (Figure 1B, bottom row). This effect is likely due to the GFP tag interfering with the C-terminal NLS which is required for nuclear transport by Karyopherin-β2. Others have shown that the insertion of GFP at the C terminus of FUS does not disrupt localization when a long linker separates the NLS and the GFP tag.^25^ To avoid the mislocalization and aggregation induced by the C-terminal GFP tag, WT, and all mutant FUS constructs were designed with an N-terminal GFP tag for subsequent studies. A GFP-only construct was also used as a control for all experiments.

Overexpression of FUS upon the transfection of FUS-GFP constructs was a potential concern because high concentration increases the condensation and aggregation propensity of FUS. To minimize overexpression, we applied the lowest possible plasmid concentration that still allows for fluorescence imaging (0.1 μg cDNA plasmid per transfection). We assessed endogenous and exogenous FUS protein levels via western blot (Figure 1D). We observed consistent endogenous FUS expression among cells transfected with WT and mutant FUS-GFP and the GFP only construct (Figure 1E). Importantly, exogenous and endogenous FUS expression was consistent among WT and all mutant FUS constructs (Figure S1), indicating a similarity in stability and expression of exogenous WT and mutant FUS constructs. When comparing endogenous and exogenous FUS protein levels, overexpression via FUS-GFP transfection resulted in a 2-fold increase of total cellular FUS protein (Figure 1F). Nevertheless, exogenous WT FUS-GFP did not cause a mislocalization of FUS nor increased aggregate formation typically seen with overexpression of FUS^23^^,^^36^^,^^37^ (Figure 1C).

The selected ALS/FLTD-linked FUS mutants were chosen to represent three classes of FUS mutants: R (R216C, R244C), G (G156E, G187S), and NLS (R495X, and P525L) mutants. R mutants of FUS exhibit defective binding to RNA leading to increased FUS aggregation, which may be reversible upon interaction with WT FUS.^34^^,^^35^ G mutants of FUS result in the formation of homotypic gel-like condensates and do not interact with WT FUS.^35^ Lastly, NLS mutants of FUS result in mislocalization from the nucleus to the cytoplasm.^29^^,^^38^ NLS mutants are the most deleterious as they result in a loss of FUS function in the nucleus coupled with a gain of toxicity in the cytoplasm. This gain of toxicity results from the FUS aggregation which is promoted by low RNA concentration in the cytoplasm.^12^ High concentrations of RNA in the nucleus have been described to help maintain the solubility of RNA-binding proteins (RBPs) such as FUS and prevent condensate formation.^12^ Therefore, a reduction in RNA concentration in cellular compartments such as the cytoplasm would promote condensate formation and could lead to aggregation.

### Localization and aggregation induced by amyotrophic lateral sclerosis/frontotemporal lobar dementia mutation and sorbitol stress

**Figure 2:**
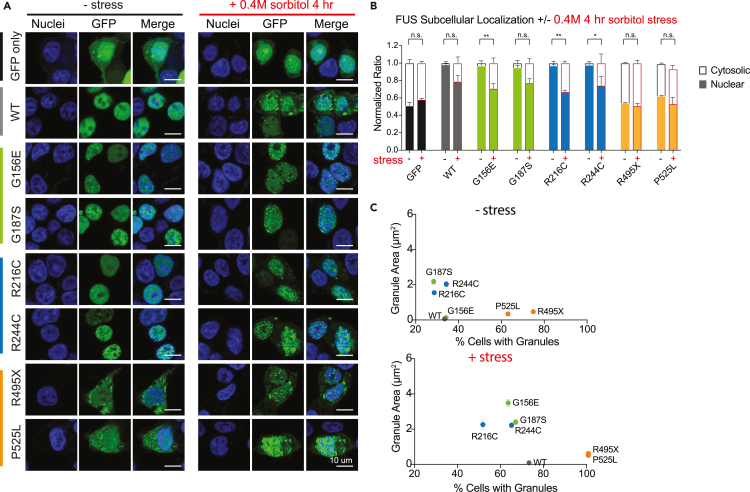
Osmotic stress induces subcellular mislocalization of FUS (A) Immunofluorescent images of SH-SY5Y cells expressing GFP only or FUS-GFP constructs under non-stressed or stressed conditions (0.4 M sorbitol 4 h treatment). Scale bar 10 μm. (B) Quantification of FUS localization in GFP only or FUS-GFP expressing cells without (−) and with (+) stress. Nuclear signal depicted in colored portion of bars, cytosolic signal depicted in transparent portion of bars. (C) Quantification of percentage of cells with granule pattern vs. granule area (μm^2^) without or with 0.4 M 4 h sorbitol stress treatment (N = 3, ∼30 cells/plasmid/condition). Data information: In (B), data are presented as mean ± SEM. ∗p ≤ 0.05, ∗∗p ≤ 0.01 (two-way ANOVA).

Cellular localization of WT and mutant FUS-GFP was determined via immunostaining. Cells were fixed and stained with nuclear dye while GFP signal from the GFP-only plasmid or FUS-GFP constructs were used to determine localization and aggregation patterns. The GFP-only plasmid control displays diffuse green signal both in the nucleus and cytoplasm as GFP can shuttle freely between both compartments (Figure 2A, top row). Under normal conditions, WT FUS displays a diffuse nuclear localization as expected (Figure 2A, second row). R and G FUS mutants display a nuclear localization, but exhibit increased punctate pattern in contrast to WT FUS, suggesting an increased propensity for condensation (Figure 2A, third-sixth rows). As expected, both NLS mutants are mislocalized to the cytoplasm due to defective NLS (Figure 2A, last two rows). The NLS mutants also display a punctate pattern in both nuclear and cytoplasmic compartments. Although the punctate pattern in the cytoplasm could be explained by the lower RNA concentration, the nuclear puncta suggest increased condensation propensity for the NLS mutants in addition to disrupted localization. The FUS granule sizes are comparable in all conditions, but the NLS mutants significantly increase the number of cells with FUS puncta (Figure 2C, top) in agreement with the higher pathogenicity of the NLS mutants. Overall, R and G mutations maintain proper localization but have increased aggregation while NLS mutants exhibit both improper localization and aggregation.

We next sought to examine the effect of stress on FUS localization and punctate formation. Osmotic stress has been known to cause prominent mislocalization of WT FUS and incorporation into stress granules.^39^^,^^40^ We asked if our FUS mutants showed the same pattern following osmotic stress. We induced osmotic stress by treating cells with 0.4 M sorbitol for 4 h. Osmotic stress resulted in the mislocalization of approximately 20% WT and 30–40% R and G mutant FUS from the nucleus to the cytosol (Figure 2A, right column). NLS mutants, which already display a ∼50% mislocalization under normal conditions, remained mislocalized to a similar degree (Figure 2B). As a control, our GFP-only construct displayed no change in localization pattern upon osmotic stress treatment. Osmotic stress also increased the granule size and cells with puncta in both WT and mutant FUS conditions (Figure 2C, bottom). We later correlate this puncta size to the cellular oligomerization status of FUS.

**Figure 3:**
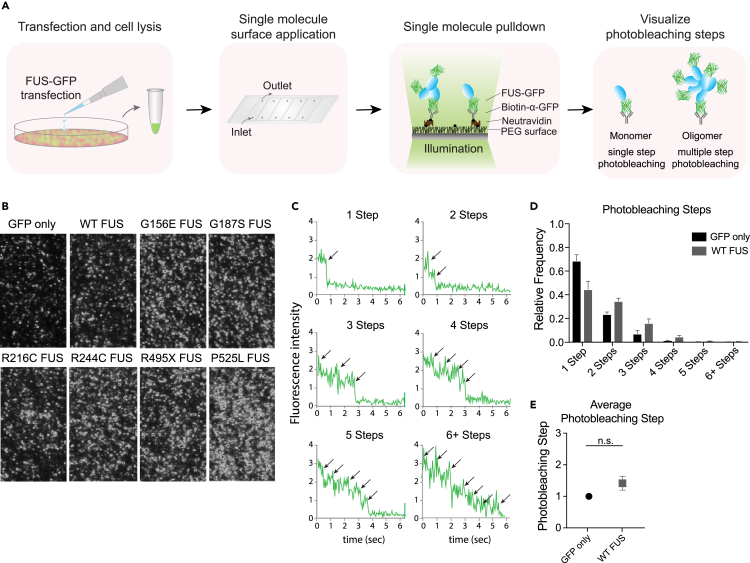
Single-molecule pulldown (SiMPull) can be used to isolate FUS complexes from cells (A) SiMPull experimental assay. (B) FUS-GFP complexes isolated from cells and captured onto the single-molecule surface are shown as individual dots on the single-molecule platform. (C) Representative photobleaching steps of individual FUS-GFP complexes. (D) Photobleaching step quantification for GFP only and wild type (WT) FUS-GFP. (E) Average photobleaching step for GFP only and WT normalized to subtract GFP-induced dimerization. GFP only (N = 1194 traces), WT (N = 1682 traces). Data information: In (D and E), data are presented as mean ± SEM. ∗p ≤ 0.01 (t-test).

Several studies have detailed the effect of oxidative stress via sodium arsenite treatment in causing cytoplasmic FUS incorporation into stress granules under mutant FUS conditions that already perturb FUS localization such as NLS mutants.^41^ Temperature has also been described as a critical regulator of phase separation.^42^^,^^43^ Therefore, we induced oxidative stress and temperature variations to cells expressing WT and NLS mutant FUS-GFP to assess the potential shift in localization or punctate pattern. Sodium arsenite treatment (1 mM for 1 h) to induce oxidative stress did not cause a robust mislocalization for either WT or mutant FUS (Figures S2A and S2D), but induced a slight increase in a punctate pattern, similar to the osmotic stress (Figures 2A and 3A). Next, we assessed the heat (42°C for 2 h) and cold (4°C for 1 h) stress. Heat (Figures S2B and S2E) and cold (Figures S2C and S2F) stress did not significantly change WT or mutant FUS except for a slight increase in punctate formation (Figures S2B and S2C) similar to all other stressors used. Based on the more drastic mislocalization phenotype displayed by sorbitol treatment of cells which better recapitulates the diseased state of ALS/FTLD-linked FUS mutants, we used sorbitol for subsequent experiments.

### Amyotrophic lateral sclerosis/frontotemporal lobar dementia -linked fused in sarcoma mutants induce higher order oligomerization

We next sought to measure the oligomeric status of cellular FUS by using the SiMPull assay. We hypothesized that FUS mutants and/or stress conditions would result in larger and more stable oligomers based on the increased condensation propensity observed at the cellular level (Figure 2) SiMPull enables efficient and robust isolation of native FUS-GFP protein complexes from cells to a single-molecule surface. Briefly, we transfect SH-SY5Y neuroblastoma cells with WT or mutant FUS-GFP constructs for ∼24 h and gently lyse the cells with NP40 cell lysis buffer to maintain FUS complexes (see STAR Methods). Cell lysates containing FUS-GFP protein are flowed onto a pre-assembled single-molecule surface coated with anti-GFP antibody (Figure 3A). GFP signals do not appear unless the surface is treated with the anti-GFP antibody, signifying a specific capture of GFP complexes (Figure 3B). The surface is illuminated with a laser beam (488 nm) and the GFP photobleaching steps (Figure 3C) are counted at each single-molecule spot to determine the number of FUS molecules in each complex. To preserve the native protein complexes, we minimized the processing time from lysing cells to setting up the imaging surface to acquiring images within 10 min.

**Figure 4:**
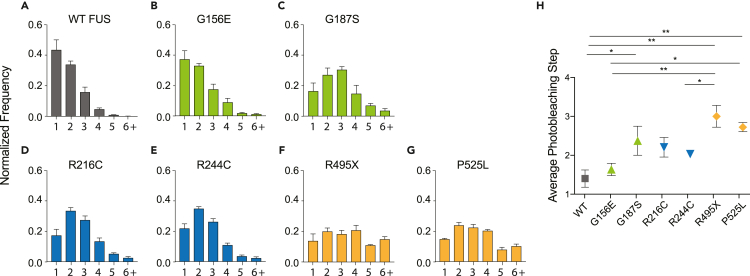
Photobleaching step distribution of WT and mutant FUS-GFP (A–G) (A) Normalized frequency of WT photobleaching step distribution, (B) G156E, (C) G187S, (D) R216C, (E) R244C, (F) R495X, and (G) P525L. (H) Average photobleaching step for WT and mutant FUS. WT (N = 1682 traces), G156E (N = 1388 traces), G187S (N = 1645 traces), R216C (N = 1616 traces), R244C (N = 1734 traces), R495X (N = 1714 traces), P525L (N = 1541 traces). Data information: In (A-H), data are presented as mean ± SEM. ∗p ≤ 0.05, ∗∗p ≤ 0.01, ∗∗∗p ≤ 0.001 (One-way ANOVA).

The GFP-only control showed successful capture of ∼70% monomers and ∼30% dimers (Figure 3D), consistent with previous reports^33^ showing a fraction of GFP forms self-dimers. The GFP oligomer pattern was used as a background to subtract dimerization states for subsequent photobleaching analysis i.e 30% of dimers in all conditions is subtracted due to GFP self-dimerization (Figure 3E). First, we compared the oligomerization status of WT to mutant FUS. WT FUS displays predominantly monomeric and dimeric states with an average photobleaching step of ∼1.5 after subtracting the GFP self-dimer fraction as stated above (Figures 3E and 4A). R and G FUS mutants display higher order oligomerization states with increased dimeric or trimeric steps with average photobleaching steps of ∼1.7–2.4 (Figures 4B–4E and 4H). Strikingly, NLS FUS mutants R495X and P525L (Figures 4F and 4G) display significantly higher oligomerization states including 4–6 steps with an average photobleaching step of ∼3 (Figure 4H). These findings reveal inherent differences in FUS complexes even at a basal level. Therefore, interactions seeding these small units of FUS likely differ among WT and mutant FUS.

### Cellular stress does not alter the oligomerization status

**Figure 5:**
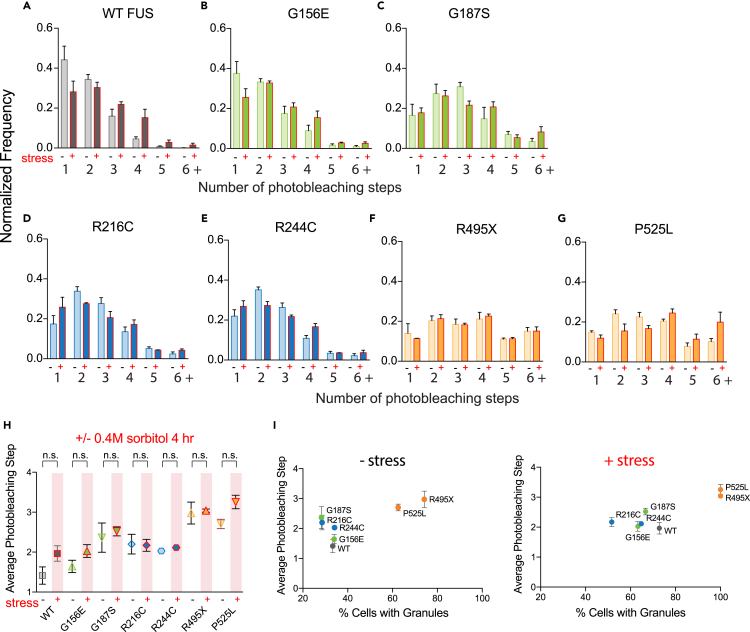
Photobleaching step distribution of WT and mutant FUS-GFP without and with osmotic stress (A–G) (A) Normalized frequency of WT photobleaching step distribution, (B) G156E, (C) G187S, (D) R216C, (E) R244C, (F) R495X, (G) P525L without (lighter colored bars) and with osmotic stress (darker, red-bordered bars). (H) Average photobleaching step for WT and mutant FUS. WT (N = 1682 traces), WT stress (N = 1180), G156E (N = 1388 traces), G156E stress (N = 1526 traces), G187S (N = 1645 traces), G187S stress (N = 1583 traces), R216C (N = 1616 traces), R216C stress (N = 1474 traces), R244C (N = 1734 traces), R244C stress (N = 1557 traces), R495X (N = 1713 traces), R495X stress (N = 1722 traces), P525L (N = 1541 traces), P525L stress (N = 1126 traces). (I) Quantification of percentage of cells with granule pattern vs. photobleaching steps without or with 0.4 M 4 h sorbitol stress treatment (N = 3, ∼30 cells/plasmid/condition). Data information: In (A–H), data are presented as mean ± SEM. ∗p ≤ 0.05 (One-way ANOVA).

The increased oligomerization observed for FUS mutants aligns with their increased propensity to form puncta at the cellular level, suggesting a potential link between oligomerization and granule formation (Figure 5I, left). To test this link, we tested if osmotic stress by sorbitol would increase the oligomers of FUS and FUS mutants since it significantly increased the puncta formation in cells (Figure 2A). Surprisingly, while WT FUS and P525L displayed a moderate increase toward higher-order oligomers with an increase in average photobleaching steps by ∼0.5 (Figures 5A and 5G), all the other FUS mutants showed similar levels of oligomers before and after the sorbitol stress although wildtype and G156E exhibited slightly increased photobleaching steps in the stressed condition (Figures 5B–5F). This result does not align with the drastic increase of FUS puncta formation under sorbitol stress shown in Figure 2 (Figure 5H). In addition, arsenite stress which induced slight aggregation of nuclear FUS did not increase photobleaching steps (Figure S3). Therefore, cellular condensation or aggregation may not be correlated with the oligomeric state of the protein or protein complexes (Figure 5I, right). Instead, oligomers that we measure by SiMPull are reflective of more fundamental aberrant interactions that occur at the molecular level for FUS mutants. Therefore, our result indicates that although the interaction between FUS monomers in FUS oligomer differs between wildtype and FUS mutants, the interaction between FUS oligomers induced by stress is likely weak, resulting in a facile dissolution into intrinsic oligomer units upon being pulled down.

### Cytosolic mislocalization alone does not cause higher fused in sarcoma oligomerization

**Figure 6:**
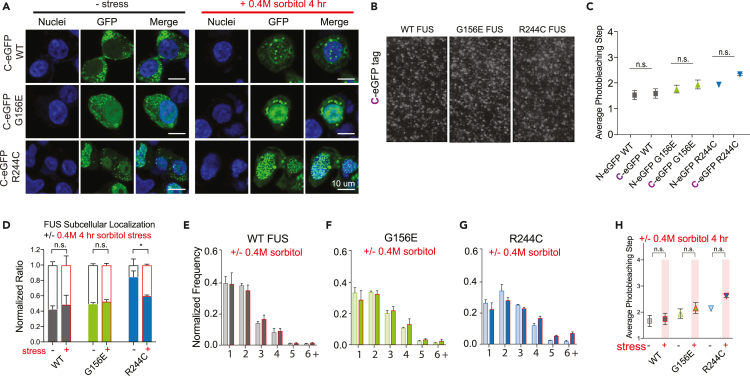
C-terminal FUS-GFP induces mislocalization but does not change oligomerization (A) Immunofluorescent images of SH-SY5Y cells expressing WT or mutant C-terminal FUS-GFP without and with osmotic stress (0.4 M sorbitol 4 h treatment). (B) FUS-GFP complexes isolated from cells and captured onto the single-molecule surface. (C) Average photobleaching steps for C-terminal WT and mutant FUS-GFP compared to N-terminal WT and mutant FUS-GFP. (D) Quantification of FUS localization without (−) and with (+) osmotic stress. (E–G) (E) Normalized frequency of WT FUS-GFP photobleaching step distribution, (F) G156E, and (G). R244C without stress (lighter colored bars) and with stress (darker, red-bordered bars). (H) Average photobleaching steps for WT and mutant FUS-GFP without and with osmotic stress. C-term WT (N = 1285 traces), C-term WT stress (N = 1469 traces), C-term G156E (N = 1510 traces), C-term G156E stress (N = 1535 traces), C-term R244C (N = 1584 traces), C-term R244C stress (N = 1420 traces), N-term WT (N = 1682 traces), N-term G156E (N = 1388 traces), N-term R244C (N = 1734 traces). Data information: In (B and D–H), data are presented as mean ± SEM. ∗p ≤ 0.05 (One-way ANOVA).

We next sought to understand if cytosolic mislocalization caused the higher oligomerization of the two NLS mutants, R495X and P252L. We took advantage of the fact that C-terminally GFP-tagged FUS results in improper cytosolic mislocalization and coacervation (Figure 1B, bottom row). We created constructs for WT, G156E, and R244C FUS with a C-terminal GFP tag to induce mislocalization even under non-stressed conditions and performed SiMPull on these constructs (Figure 6A, left panel). C-term constructs did not result in an increase in oligomerization relative to N-term constructs (Figures 6B and 6C). Osmotic stress only slightly increased mislocalization (Figure 6A, right panel, 6D) since FUS was already mislocalized due to the C-term GFP tag. We note that the R244C displayed less mislocalization than the WT possibly because the aggregated state of R244C in the nucleus prevented mislocalization into the cytoplasm. In addition, stress did not increase the oligomerization of C-term FUS constructs (Figures 6E–6H). Therefore, cytosolic localization itself is not sufficient to promote higher order oligomerization and the increased oligomerization pattern of FUS mutants, particularly NLS mutants, is potentially due to other complex interactions.

### Fused in sarcoma oligomers are highly stable and resistant to dissolution

Multivalent interactions are known to drive LLPS of FUS and other RBPs.^14^^,^^15^ Electrostatic,^20^^,^^44^ hydrophobic,^45^^,^^46^^,^^47^ protein, and RNA^12^^,^^34^^,^^35^ interactions are thought to underlie the formation of ribonucleoprotein (RNP) granules. To better understand the interactions maintaining WT FUS or driving enhanced oligomerization of mutant FUS, we systematically perturbed these multivalent interactions with dissolving agents and compared the number of photobleaching steps before and after dissolving agents. We expect that effective dissolving agents will disrupt basal FUS-GFP complexes, resulting in primarily monomeric states of WT and mutant FUS.

**Figure 7:**
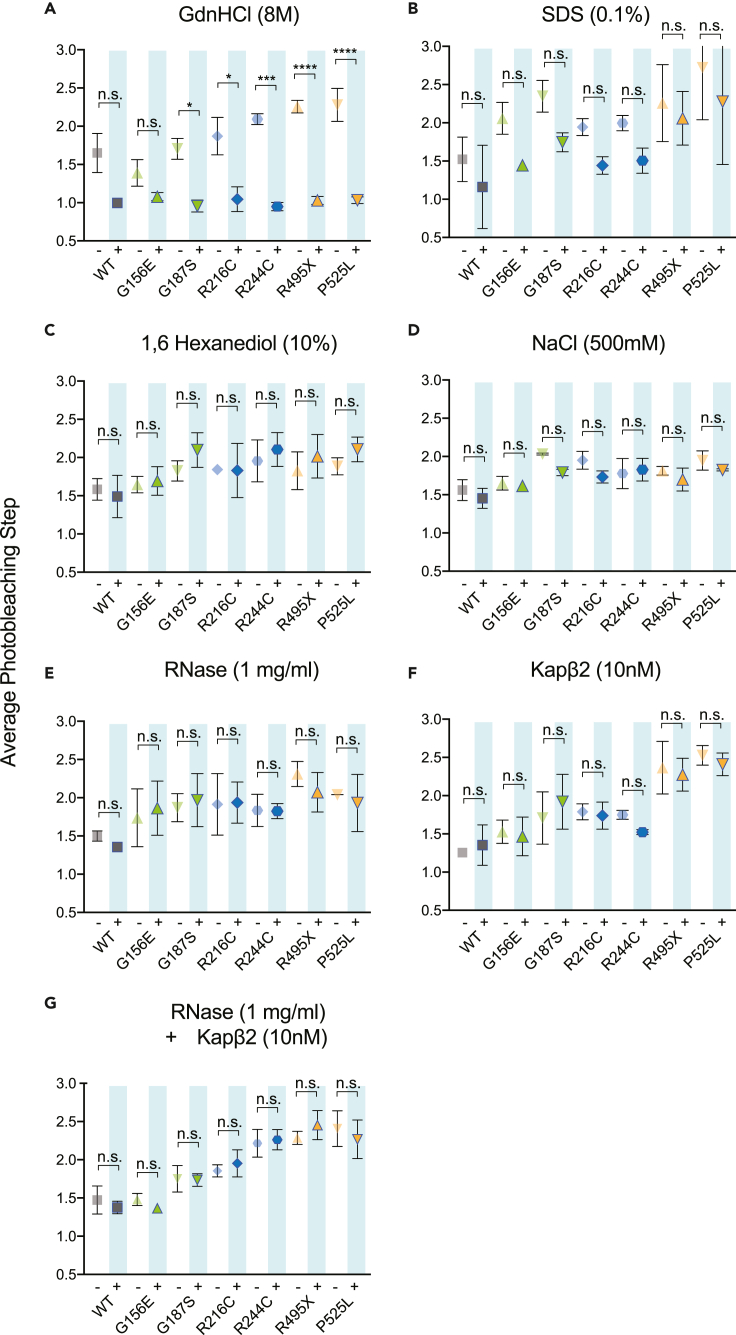
WT and mutant FUS-GFP are stable and resistant to dissolution (A–F) (A) Normalized difference of WT and mutant FUS photobleaching step distribution before and after dissolution with 8 M guanidine hydrochloride (GdnHCl), (B) 0.1% sodium dodecyl sulfate (SDS), (C) 10% 1,6 hexanediol, (D) 500 mM sodium chloride (NaCl), (E) 1 mg/mL RNase, and (F) 10 nM karyopherin β2 (Kapβ2). WT, G156E, G187S, R216C, R244C, R495X, P525L before and after dissolving agent (N = ∼900 traces each). Data information: In (A–G), data are presented as mean ± SEM. ∗p ≤ 0.05, ∗∗p ≤ 0.01, ∗∗∗p ≤ 0.001 (One-way ANOVA).

As a control, a strong protein denaturant (8 M guanidine hydrochloride (GdnHCl) and protein denaturant (0.1% sodium dodecyl sulfate (SDS)) was applied to dissolve FUS-GFP complexes and perturb oligomeric interactions. GdnHCl treatment resulted in a markedly increased monomeric population of WT and mutant FUS following a 5 min incubation (Figure 7A). SDS resulted in a mild increase in the monomeric population of WT and mutant FUS (Figure 7B). We next applied 10% 1,6-hexanediol (Figure 7C), 500 mM NaCl (Figure 7D), and 1 mg/mL RNase A (Figure 7E) to probe the importance of hydrophobic, electrostatic, and RNA-dependent interactions, respectively. Strikingly, none of these treatments significantly disrupted the oligomers of WT or mutant FUS (Figure S4). Thus, these basal-level FUS oligomers are highly stable nano clusters that are not maintained by the same weak, multivalent interactions characteristic of condensates. Even WT FUS oligomers did not decrease or trend toward a predominantly monomeric pool of FUS, highlighting the presence of stable basal FUS oligomers that are resistant to dissolution.

We next assessed whether Karyopherin beta 2 (Kapβ2) could dissolve FUS oligomers. KapB2 is a known nuclear importin of FUS which binds the NLS of FUS and helps to promote solubility by preventing interactions with FUS or other RBPs.^48^^,^^49^^,^^50^^,^^51^ Kapβ2 also prevents phase separation and incorporation of FUS into condensates by importing FUS back into the nucleus where RNA can buffer phase separation.^49^^,^^51^ Mutations in FUS that prevent FUS-Kapβ2 interactions result in enhanced FUS aggregation and pathological inclusions.^29^^,^^38^ Because Kapβ2 binds the NLS of FUS, mutations in this region are especially prone to aggregation. Thus, we expected Kapβ2 may be able to bind and help disaggregate WT and other mutant FUS oligomers, although we expected less of an effect for NLS mutants, R495X and P525L. Surprisingly, we found Kap β2 did not solubilize WT or mutant FUS at 10–1000 nM Kapβ2 and roughly equimolar or higher isolated FUS-GFP on the single-molecule surface, (Figure 7F). Kapβ2 was ineffective in dissolving WT or mutant FUS oligomers, which may be due to the FUS-FUS or FUS-RBP interactions that mask the NLS domain. In addition, Kapβ2 combined with RNase A (Figure 7G) to perturb FUS-FUS, FUS-RBP, and FUS-RNA interactions was unable to dissolve oligomers.

Overall, dissolution experiments revealed that our isolated FUS-GFP oligomers captured from cells in their native contexts were highly stable and resistant to dissolution except in strong protein denaturation conditions.

## Discussion

Mislocalization and aggregation of many RBPs such as FUS and TDP43 are major hallmarks of degenerating neurons.^52^ The same pattern was recapitulated in cell-based studies in which disease-linked mutant proteins or stress conditions were applied. While mislocalization has been induced in NLS mutants or under severe stress or aged conditions, mislocalization has not previously been correlated with oligomerization pattern. Condensation or aggregation in aged or severed ALS/FTLD-linked FUS mutants has also been observed *in vitro* and in cells but has not been correlated with oligomerization. Other studies also fail to capture basal RNP complexes in a cellular context and focus either on full condensates in cells or RNP complexes with minimal RNA and protein components using purified protein *in vitro*.

SiMPull enables the capture of native FUS complexes from cells within minutes using minimal cell lysate. This promotes the preservation of multivalent interactions maintaining small FUS complexes. With SiMPull, we were able to detect the oligomerization patterns of WT and ALS/FTLD-linked FUS. We confirm that FUS WT exists primarily as monomers in cells. Strikingly, we observed an increase in FUS oligomerization states with NLS mutants R495X and P525L. Such increased oligomerization seen in NLS mutants is not solely due to the mislocalization since the C-terminally GFP-tagged FUS WT which is also mislocalized does not exhibit increased oligomerization. FUS mutants G187S, R216C, and R244C also showed increases in oligomerization states compared to WT FUS. The increased oligomerization under sorbitol stress we report here is likely underestimated because a considerable amount of FUS remained soluble i.e as monomers or dimers in the nucleus which was mixed with the higher oligomers of cytoplasmic condensates for the experiment.

FUS complexes captured by SiMPull reveal the oligomerization state of WT and mutant FUS. Although we cannot directly relate the oligomerization to cellular toxicity, we show that the oligomer state of FUS mutants is highly correlated with the cellular features often associated with neurodegenerative diseases including the level of FUS granule formation, granule size, and mislocalization (Figure S5). However, we note that these complexes may contain other components such as proteins and RNA that maintain the stability of oligomers. In the future, we aim to discern the components of isolated complexes by tagging other proteins or RNAs with different fluorophores. This will further enable us to visualize the mixing of WT and mutant FUS with each other and with other molecules, providing insight into the composition and resulting properties of such oligomers.

Basal FUS oligomers rapidly isolated from cells and preserved in their native contexts gives us an indication of how these units differ in oligomer states and which interactions may underlie the formation of these oligomers. Differences between WT and mutant FUS oligomers may indicate potential issues with ALS/FTLD-linked FUS mutants whose multimerization at a single-molecule level is already perturbed and underlies the formation of pathological FUS aggregates. This suggests that SiMPull enables the isolation of basal FUS oligomeric units from their native states. Thus, SiMPull captures FUS oligomers as the basic building units of FUS condensates and enables us to further study the interactions that promote FUS oligomerization at a single-molecule level. While we are not pulling down FUS condensates, we are able to isolate native, soluble FUS oligomers in their basal states. There already exists a difference between WT and ALS/FTLD-linked FUS mutants in their oligomeric states under normal conditions. Interestingly, stress does not increase FUS oligomerization. Therefore, stress potentially acts on FUS condensates by promoting FUS aggregation via multivalent interactions among basal oligomers that lead to the formation of pathological aggregates over time.

### Limitations of the study

The FUS oligomers that we pulldown from cell lysate and apply to SiMPull measurement may not capture the full extent of cellular oligomers of FUS as we may lose some loosely bound proteins during the process i.e obtaining cell lysate and making dilutions required for the SiMPull experiment. The ectopic overexpression of FUS-GFP may impact the oligomerization status of FUS although the same condition applies to all measurements (WT, FUS mutants, stress and so forth) equally.

## STAR★Methods

### Key resources table

**Table:** 

REAGENT or RESOURCE	SOURCE	IDENTIFIER
**Antibodies**		

Rabbit anti-GFP antibody biotin conjugated	Rockland	600-406-215; RRID: AB_828168
Mouse monoclonal FUS/TLS antibody	Santa Cruz Biotech	sc-47711; RRID: AB_2105208
Mouse anti-alpha Tubulin antibody [DM1a]	Abcam	ab7291; RRID: AB_2241126
Goat Anti-Rabbit IgG H&L (Alexa Fluor 568)	Abcam	A-11011; RRID: AB_143157
Goat Anti-Mouse IgG H&L (Alexa Fluor 647)	Abcam	A-21235; RRID: AB_2535804
Goat anti-Mouse IgG (H+L) Secondary Antibody, HRP	Thermo Fisher	31430; RRID: AB_228307
Goat anti-Rabbit IgG (H+L) Secondary Antibody, HRP	Thermo Fisher	31460; RRID: AB_228341

**Bacterial and virus strains**		

NEB Turbo Competent *E. coli* (High Efficiency)	New England Biolabs	C2984H

**Chemicals, peptides, and recombinant proteins**		

cOmplete™ Protease Inhibitor Cocktail	Roche	11697498001
Sodium (meta)arsenite (>90%)	Millipore Sigma	S7400-100G
D-Sorbitol	Millipore Sigma	S1876-100G
Bovine Serum Albumin (BSA)	VWR	97061-420
Ribonuclease A from bovine pancreas	Millipore Sigma	R6513-50MG
1,6-Hexanediol,99%	Millipore Sigma	240117-50G
Guanidine hydrochloride	Millipore Sigma	G3272-100G
NP40 Cell Lysis Buffer	Thermo Fisher	FNN0021
Formaldehyde solution	Millipore Sigma	47608-1L-F
Hoechst 33342 Solution (20 mM)	Thermo Fisher	62249
Pierce™ ECL Western Blotting Substrate	Thermo Fisher	32109
Karyopherin β2	Guo et al., 2018	N/A
DMEM, high glucose, no glutamine	Thermo Fisher	11960-044
Fetal Bovine Serum, certified, United States	Thermo Fisher	16000-044
Sodium Bicarbonate 7.5% solution	Thermo Fisher	250-80-094
Sodium Pyruvate (100 mM)	Thermo Fisher	113-60-070
L-Glutamine (200 mM)	Thermo Fisher	250-30-081
Penicillin-Streptomycin (10,000 U/mL)	Thermo Fisher	15-140-122

**Experimental models: Cell lines**		

Human: SH-SY5Y	ATCC	Cat# CRL-2266; RRID: CVCL_0019

**Recombinant DNA**		

pcDNA3.1(+)-N-eGFP	Genscript	N/A
pFUS-WT-GFP_pcDNA3.1(+)-C-eGFP	Genscript	N/A
pFUS-G156E-GFP_pcDNA3.1(+)-C-eGFP	Genscript	N/A
pFUS-R244C-GFP_pcDNA3.1(+)-C-eGFP	Genscript	N/A
pFUS-WT-GFP_pcDNA3.1(+)-N-eGFP	Genscript	N/A
pFUS-G156E-GFP_pcDNA3.1(+)-N-eGFP	Genscript	N/A
pFUS-G187S-GFP_pcDNA3.1(+)-N-eGFP	Genscript	N/A
pFUS-R216C-GFP_pcDNA3.1(+)-N-eGFP	Genscript	N/A
pFUS-R244C-GFP_pcDNA3.1(+)-N-eGFP	Genscript	N/A
pFUS-R495X-GFP_pcDNA3.1(+)-N-eGFP	Genscript	N/A
pFUS-P525L-GFP_pcDNA3.1(+)-N-eGFP	Genscript	N/A

**Software and algorithms**		

MATLAB and IDL scripts	http://physics.illinois.edu/cplc/software This manuscript	N/A
Adobe Illustrator	Adobe (https://www.adobe.com/products/illustrator.html)	N/A
Prism 7	GraphPad (https://www.graphpad.com/scientific-software/prism/)	N/A
ImageJ (Fiji)	NIH (https://imagej.nih.gov/ij/)	N/A
Zen Blue	Zeiss	N/A

**Other**		

4–20% Mini-PROTEAN® TGX™ Precast Protein Gels, 15-well, 15 μL	Bio-Rad	4561096
Nitrocellulose membranes, roll, pore size 0.45 mm	VWR	10-6000-02
Novex™ 10X Tris-Glycine SDS running buffer	Thermo Fisher	LC26754
10x Tris/Glycine Buffer for Western Blots and Native Gels	Bio-Rad	1610772
E.Z.N.A.® Plasmid DNA Mini Kit I, (V-spin)	Omega Bio-Tek	D6943-01

### Resource availability

#### Lead contact

Further information and requests for resources and reagents should be directed to and will be fulfilled by the Lead Contact, Sua Myong (smyong1@jhu.edu).

#### Materials availability

All unique reagents generated in this study are available from the lead contact without restriction.

#### Code availability

MATLAB code from this manuscript can be downloaded from Github: (https://github.com/Myong-Lab). IDL (http://www.exelisvis.co.uk/ProductsServices/IDL.aspx), MATLAB (https://www.mathworks.com/), Adobe (https://www.adobe.com/products/photoshop.html), and Prism7 (https://www.graphpad.com/scientific-software/prism/) software with academic or individual licenses can be obtained from their respective software companies. ImageJ is an open-source program available from the NIH (https://imagej.nih.gov/ij/).

### Experimental model and subject details

#### Mammalian cell culture

Female human SH-SY5Y neuroblastoma cells (ATCC) were grown at 37°C with 5% CO2 in a DMEM solution supplemented with 10% FBS, 2 mM glutamate, 100 mg/mL Pen-Strep, 0.15% sodium bicarbonate, and 1 mM sodium pyruvate. Cell lines were periodically tested with a mycoplasma-detecting test. A T75 flask with 10 mL DMEM was used to grow and passage cells, and cells were washed twice with 1X distilled PBS before trypsinizing with 0.05% trypsin. The trypsin was neutralized by 1:10 addition of DMEM. Cells were diluted to 1:10–1:20 in new flasks. For long-term storage, SH-SY5Y cells were trypsinized and DMSO was added to a final concentration of 5% (v/v). Cells were first frozen at 80°C before being transferred to liquid nitrogen for long-term storage.

### Method details

#### Plasmid generation

WT and mutant FUS plasmids were obtained from GenScript. FUS cDNA was inserted into pcDNA3.1+N-eGFP or pcDNA3.1+C-eGFP backbone. pcDNA3.1+N-eGFP backbone without gene insertion was also used for a control in experiments where a GFP only plasmid is denoted.

#### Mammalian cell transfection

Transfections were performed via calcium phosphate transfection using 2.5 M CaCl_2_ and 2X HEPES buffered saline pH 7.0 (274 mM NaCl, 10 mM KCl, 1.4 mM Na_2_HPO_4_·7H_2_O, 15 mM dextrose, 42 mM HEPES). WT and mutant FUS constructs were transfected at minimal DNA concentrations to minimize FUS aggregation or mislocalization due to overexpression.

#### Western blot

Cells transfected for ∼24 hrs were lysed with NP40 cell lysis buffer supplemented with cOmplete^TM^ EDTA-free protease inhibitor cocktail for 10 min on ice. Cell lysates were then centrifuged at 13,000 x g for 10 min at 4°C to obtain a soluble protein pool. 4X Laemmli buffer (4% SDS, 20% glycerol, 0.004% bromophenol blue, 0.125 M Tris-HCl, 10% beta-mercaptoethanol) was added to samples. Samples were subsequently heated to 100°C for 5 min prior to loading samples on a 4–20% polyacrylamide gel and running in Novex^TM^10X Tris-Glycine SDS running buffer diluted to 1X. Gel was then transferred to nitrocellulose membrane for antibody probing. Primary antibodies were diluted in blocking buffer (5% milk, 1X PBS-Tween 20) and blot was incubated overnight in 4°C. Secondary antibodies were diluted in blocking buffer and blot was incubated 1–2 hrs at RT. Washing steps in between antibody incubations were performed with 1X PBST. Pierce^TM^ ECL Western Blotting Substrate was applied to blots for chemiluminescent detection and subsequent analysis.

#### Immunocytochemistry

Cells were grown on glass bottom chambers prior to immunostaining. Cells were fixed ∼24 hrs after transfection in formaldehyde diluted to 4% in 1X PBS for 10 min at RT. Cells were permeabilized and blocked in buffer (0.3% Triton^TM^ X-100 and 1% BSA in 1X PBS) for 10 min. Cells were incubated with primary antibodies in buffer overnight at 4°C. Secondary antibodies combined with Hoechst dye were diluted in buffer and incubated for 1 hr at RT. Washing steps after antibody incubation were performed with 1X PBS. Cells were maintained in 1X PBS at 4°C after staining and imaged the same or next day via confocal microscopy.

#### Confocal microscopy

Confocal microscopy was performed at the Integrated Imaging Center at the Johns Hopkins Homewood Campus with a Zeiss LSM 700 microscope equipped with lasers for 405, 488, 568, and 647 nm excitation. Images were acquired using four-fold frame averaging with a 40 × 1.4 oil objective. The same laser and acquisition settings were maintained for imaging all samples within one experiment.

#### Single molecule pulldown

For single molecule pulldown, a methoxy polyethylene glycol (PEG) coated microscope slide and coverslip were prepared following a previously described method.^53^ ∼2% biotinylated PEG was added during slide preparation to allow for specific immobilization of biotinylated molecules to the surface. Holes were drilled on opposite ends of the slide to allow for rapid flow of cell lysate and exchange of solution. Flow chambers were constructed using double sided tape to seal the coverslip to the slide, and edges of the slide and coverslip were secured with epoxy. ∼20 μL of solution is enough to saturate each chamber. Once prepared, 30 μL neutravidin was flowed into the chamber at a 20 μg/μL dilution and incubated for ∼2 min 50 μL of T50 buffer (10 mM Tris-Cl pH 8.0, 50 mM NaCl) was flowed through to wash off unbound neutravidin, then 50 μL biotin conjugated anti-GFP was flowed onto the surface at a 1:200 dilution for ∼2 min followed by another 50 μL T50 buffer wash. Cells transfected for ∼24 hrs were lysed with NP40 cell lysis buffer supplemented with cOmplete^TM^ EDTA-free protease inhibitor cocktail for 1 min. Cell lysates containing soluble protein were then diluted 1:100–1:10,000 in T50 buffer and 50 μL of diluted cell lysate was flowed onto the single molecule surface to obtain a surface density of ∼100–300 molecules in the 2,500 μm^2^ imaging area. A 50 μL T50 buffer wash was performed to remove unbound protein. A prism type total internal reflection fluorescence microscope equipped with an electron-multiplying charge-coupled device was used for single molecule imaging. GFP-tagged proteins were excited at 488 nm. All single molecule experiments were performed at room temperature. Single molecule fluorescence time traces of surface immobilized GFP-tagged proteins were manually quantified for number of photobleaching steps. Cell lysates were diluted accordingly to obtain ∼100–300 molecules on the single molecule imaging surface to avoid oversaturation of GFP molecules.

#### Dissolution experiments

Using the same single molecule pulldown protocol described above, cell lysates were isolated for imaging on the single molecule surface. Single molecule imaging was performed to obtain photobleaching steps for GFP-only, WT, and mutant FUS complexes. Then, 50 μL dissolving agent was applied to the chamber and incubated for ∼5 min. Two washes of 50 μL T50 buffer was used to remove dissolving agent and any unbound, dissolved GFP-only or FUS-GFP protein. Single molecule imaging was performed again to obtain photobleaching steps following dissolving agent addition.

### Quantification and statistical analysis

#### Image analysis

Confocal images were acquired in ZEN (Zeiss). Images were processed using ImageJ/FIJI software. Artificial colors were applied for individual channels as depicted in figures.

#### Quantification of FUS localization in cells

FUS subcelluar localization quantification was performed in ImageJ. A boundary was drawn around total GFP signal within each cell. Nuclear GFP signal was then subtracted to obtain cytosolic GFP signal. Nuclear and cytosolic GFP signal were divided by total cellular GFP signal to obtain ratios.

#### Quantification of FUS granule area

FUS granule area quantification was performed in ImageJ. A boundary was drawn around each individual granule to obtain the area.

#### Quantification of photobleaching steps

Photobleaching steps were analyzed via a MatLab script Trace_Viewer_Categorizer which enables stepwise traces to be visualized. Photobleaching steps were quantified manually and counted if a stepwise decrease in fluorescent signal intensity was observed.

## Data Availability

The source data for all the relevant figures are provided with this paper. Source data are provided with this paper.
