# Supplementary material for: Nucleation and dissolution mechanism underlying amyotrophic lateral sclerosis/frontotemporal lobar dementia-linked fused in sarcoma condensates
Source: iScience. 2023 Mar 31;26(4):106537. doi: 10.1016/j.isci.2023.106537 (PMC10139993; doi:10.1016/j.isci.2023.106537)
Supplement: Document S1. Figures S1–S5 [file mmc1.pdf]

iScience, Volume *26*

Supplemental information

Nucleation and dissolution mechanism underlying amyotrophic lateral sclerosis/frontotemporal lobar dementia-linked fused in sarcoma condensates

Nathalie A. Djaja, Matthew T. Chang, Freya R. Beinart, Vivian M. Morris, Laura R. Ganser, and Sua Myong

Supplementary Figure 1

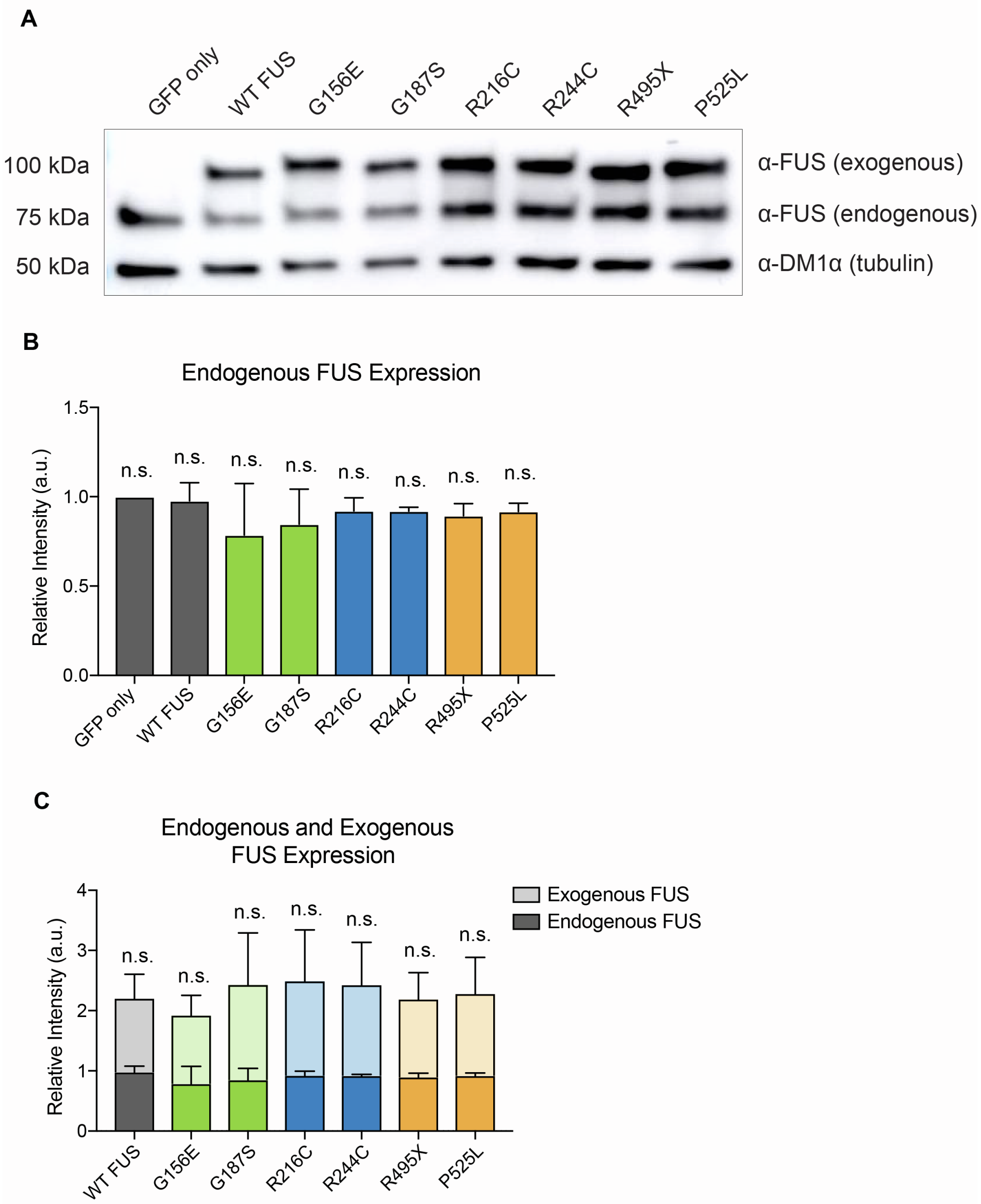

Supp Figure 1 – [Exogenous expression of WT and mutant FUS-GFP is consistent], Related to Figure 1

A. Western blot of GFP only, WT, or mutant FUS-GFP expressing cells probed with α-FUS and α-tubulin antibody.

B. Endogenous FUS expression among GFP only, WT, or mutant FUS-GFP is consistent (N = 3).

C. Exogenous FUS expression among GFP only, WT, or mutant FUS-GFP is consistent and increases total cellular FUS level one-fold (N = 3).

Data information: In (B-C) data are presented as mean ± SD. *P≤0.05 (One-way ANOVA)

Supplementary Figure 2

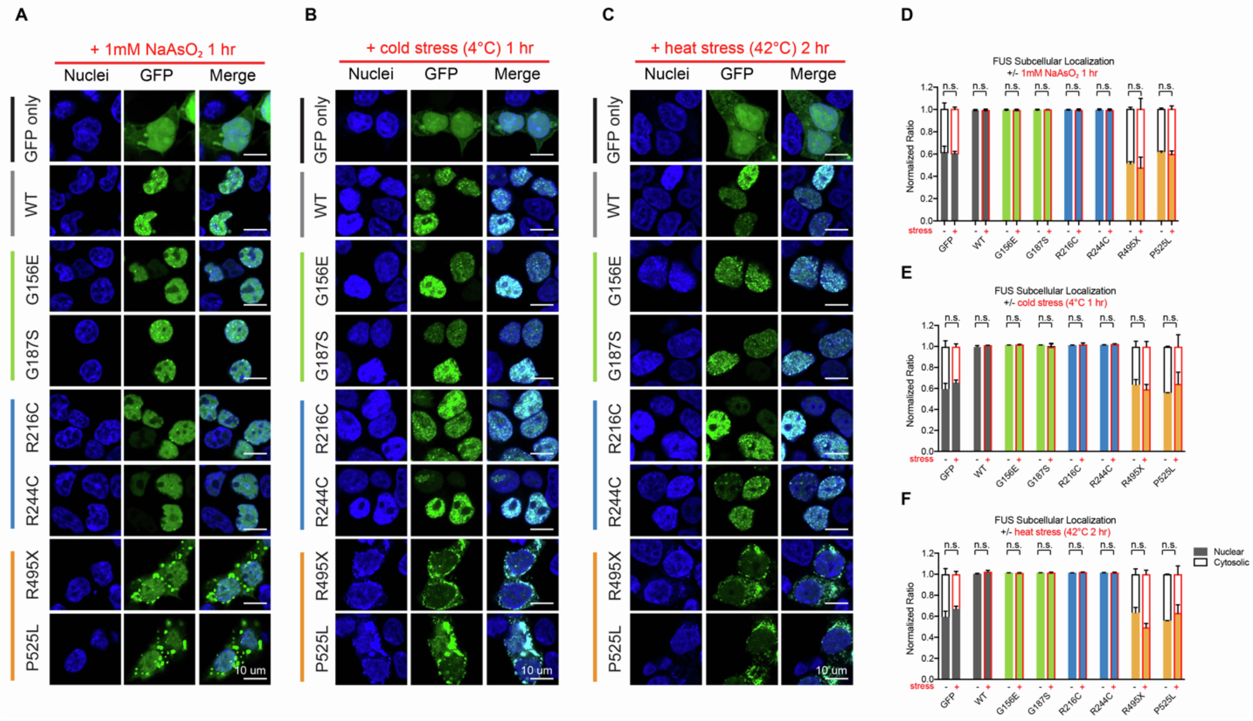

Supp Figure 2 – [Oxidative, heat, or cold stress does not alter FUS subcellular localization], Related to Figure 2

A. Immunofluorescent images of SH-SY5Y cells expressing FUS-GFP under oxidative stress (1mM $NaAsO_2$ 2 hr treatment), **B**. cold stress (4°C 1 hr treatment), or **C**. heat stress (42°C 2 hr treatment). Scale bar 10 μm.

D. Quantification of FUS localization in GFP only or FUS-GFP expressing cells without (-) and with (+) oxidative stress, **E**. cold stress, or **F**. heat stress treatment.

(N = 3, ~30 cells/plasmid/condition)

Data information: In (D-F), data are presented as mean ± SEM. *P≤0.05 (Two-way ANOVA)

Supplementary Fig 3

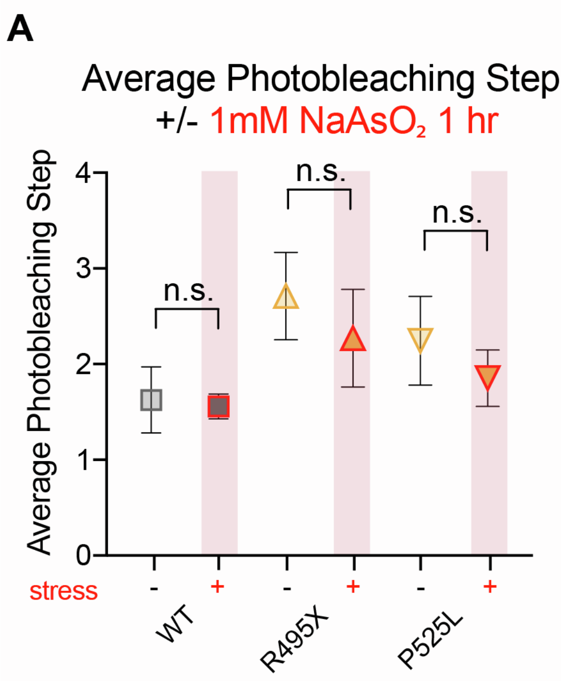

Supp Figure 3 – [Oxidative stress does not alter FUS-GFP oligomerization], Related to Figure 5

A. Average photobleaching step for WT, R495X, and P525L FUS-GFP without (lighter symbols) or with (darker, red-bordered symbols).

WT (N = 264 traces), WT stress (N = 265 traces), R495X (N = 300 traces), R495X stress (N = 272 traces), P525L (N = 300 traces), P525L stress (N = 272 traces)

Data information: In (A) data are presented as mean ± SEM. *P≤0.05 (One-way ANOVA)

Supplementary Figure 4

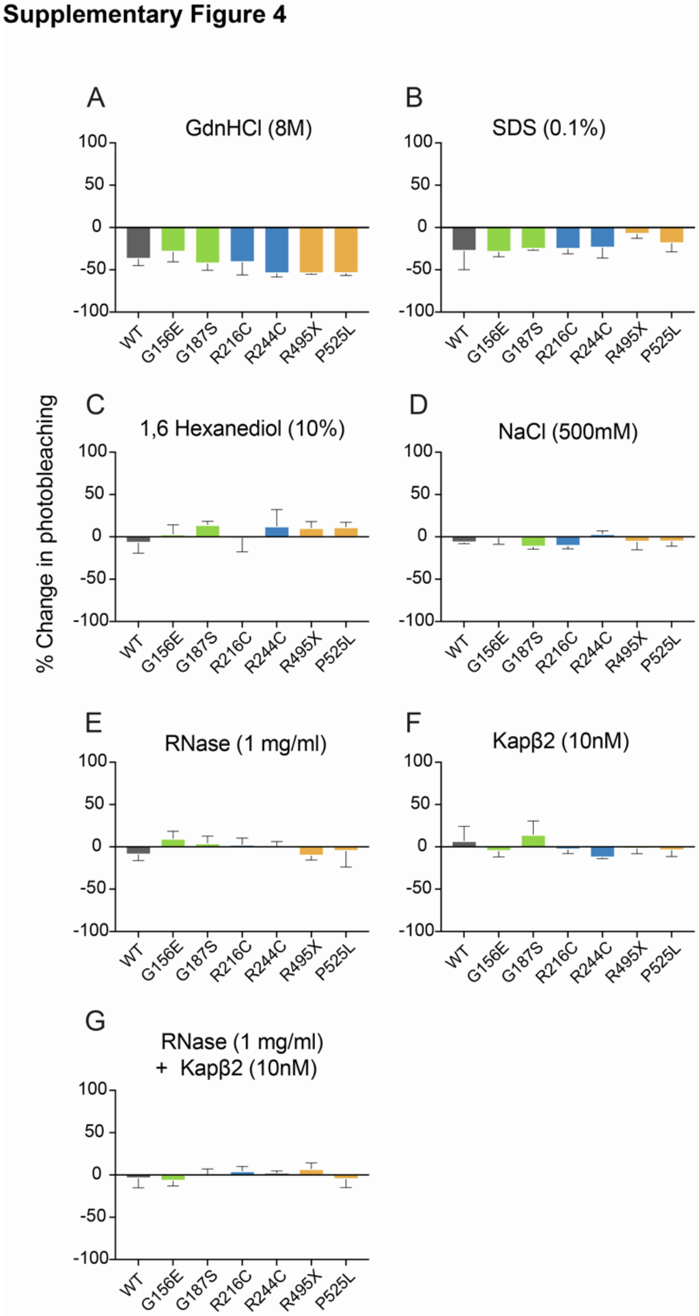

Supp Figure 4 – [WT and mutant FUS-GFP are stable and resistant to dissolution], Related to Figure 7

A. Photobleaching steps before and after dissolution with 8M guanidine hydrochloride (GdnHCl), **B**. 0.1% sodium dodecyl sulfate (SDS), **C**. 10% 1,6 hexanediol, **D**. 500mM sodium chloride (NaCl), **E**. 1 mg/mL RNase, **F.** 10nM karyopherin β2 (Kapβ2), and **G**. 1 mg/mL RNase + 10nM karyopherin β2 (Kapβ2).

WT, G156E, G187S, R216C, R244C, R495X, P525L before and after dissolving agent (N = ~900 traces each)

Data information: In (A-F), data are presented as mean ± SEM

Supplementary Figure 5

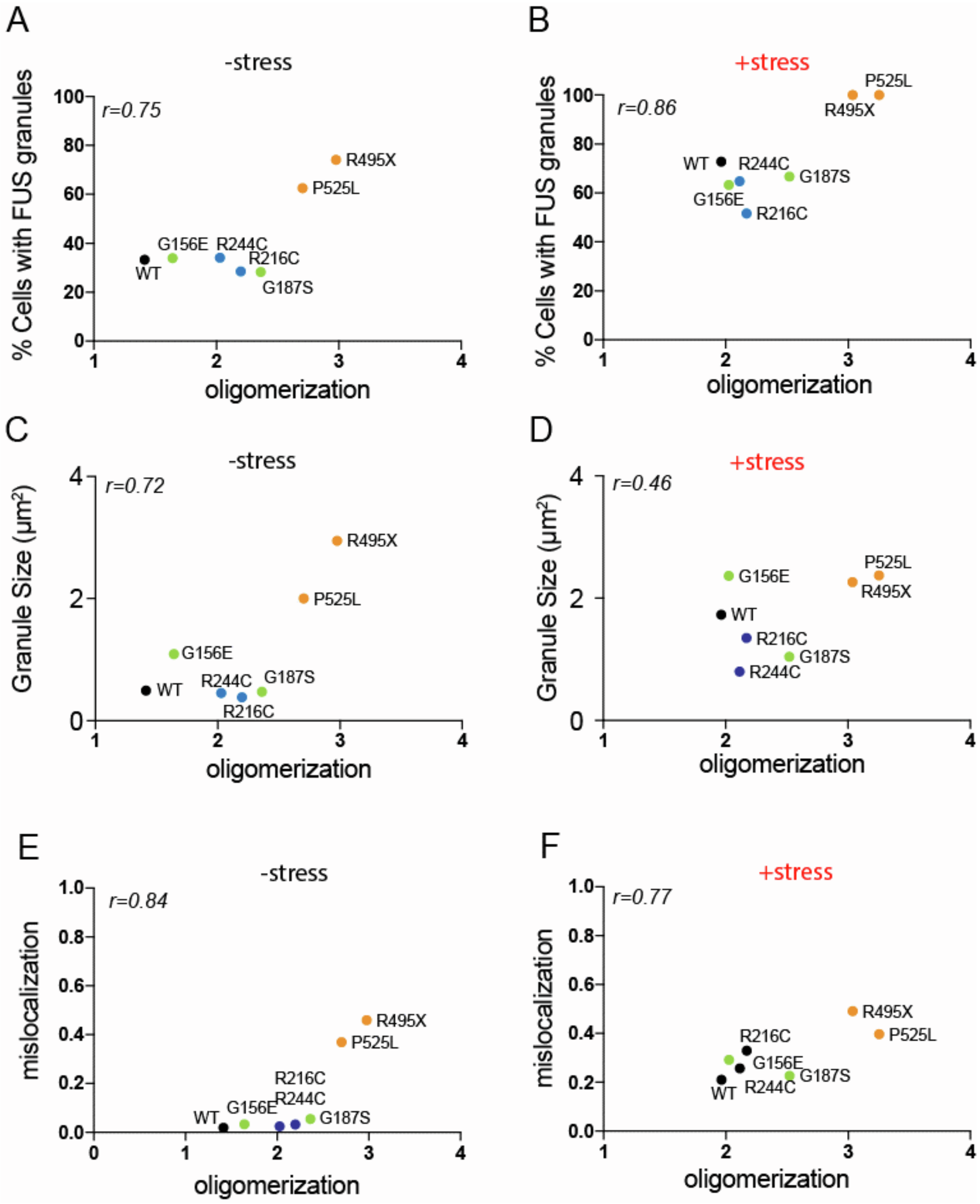

Supp Figure 5 – [Oligomerization correlated to FUS granule pattern and mislocalization], Related to Figure 2 and 5

A. FUS oligomerization (x-axis) is correlated to the percent (y-axis) of cells containing FUS granules without and **B.** with stress. **C.** FUS oligomerization (x-axis) is correlated to granule size (y-axis) without and **D.** with stress. **E.** FUS oligomerization (x-axis) is correlated to the cytosolic mislocalization (y-axis) of FUS without and **F.** with stress.
WT, G156E, G187S, R216C, R244C, R495X, P525L (N = ~1000 traces each, N = 3, ~30 cells/plasmid/condition)
